# Automated Distal Radius and Ulna Skeletal Maturity Grading from Hand Radiographs with an Attention Multi-Task Learning Method

**DOI:** 10.3390/tomography10120139

**Published:** 2024-11-28

**Authors:** Xiaowei Liu, Rulan Wang, Wenting Jiang, Zhaohua Lu, Ningning Chen, Hongfei Wang

**Affiliations:** 1 School of Computing and Artificial Intelligence, Shandong University of Finance and Economics, Jinan 250000, China; 2 Shenzhen Institute of Advanced Technology, Chinese Academy of Sciences, Shenzhen 518000, China; 3 Department of Diagnostic Radiology, The University of Hong Kong, Hong Kong 999077; 4 Department of Orthopedic Surgery, The Seventh Affiliated Hospital, Sun Yat-Sen University, Shenzhen 518000, China; 5 Shenzhen Key Laboratory of Bone Tissue Repair and Translational Research, Shenzhen 518000, China; 6 Department of Orthopaedics and Traumatology, The University of Hong Kong, Hong Kong 999077

**Keywords:** bone age, hand-wrist X-ray, scoliosis, deep learning, classification, segmentation

## Abstract

**Background:** Assessment of skeletal maturity is a common clinical practice to investigate adolescent growth and endocrine disorders. The distal radius and ulna (DRU) maturity classification is a practical and easy-to-use scheme that was designed for adolescent idiopathic scoliosis clinical management and presents high sensitivity in predicting the growth peak and cessation among adolescents. However, time-consuming and error-prone manual assessment limits DRU in clinical application. **Methods**: In this study, we propose a multi-task learning framework with an attention mechanism for the joint segmentation and classification of the distal radius and ulna in hand X-ray images. The proposed framework consists of two sub-networks: an encoder–decoder structure with attention gates for segmentation and a slight convolutional network for classification. **Results:** With a transfer learning strategy, the proposed framework improved DRU segmentation and classification over the single task learning counterparts and previously reported methods, achieving an accuracy of 94.3% and 90.8% for radius and ulna maturity grading. **Findings:** Our automatic DRU assessment platform covers the whole process of growth acceleration and cessation during puberty. Upon incorporation into advanced scoliosis progression prognostic tools, clinical decision making will be potentially improved in the conservative and operative management of scoliosis patients.

## 1. Introduction

Skeletal maturity is a measure of physiological development status and remaining growth potential for immature children and adolescents during their pubertal growth period [1,2]. It forms an important part of the diagnosis and management guidelines for adolescent growth and endocrine disorders [3,4]. For example, significant discrepancies between an individual’s bone age and their chronological age could suggest the presence of a growth disorder [5]. Supplemental hormone therapy for growth abnormality relies on skeletal maturity assessment in the decision of when to start and stop therapy [6,7]. In particular, the estimation of growth spurt stage and remaining growth potential carries an important implication in idiopathic scoliosis management strategy, because rapid scoliotic curve deterioration mainly occurs around growing peaks [8].

Assessment of skeletal maturity is generally performed with a non-dominant hand and wrist radiograph, covering the distal radius and ulna and all the fingers [9,10]. Radiographic imaging of hand and wrist regions can capture more bones but with minimal radiation [11]. X-ray imaging of the knee, elbow, cervical vertebrae, and pelvis are also reported to be utilized for bone age assessment [12,13,14,15]. Morphology features of these bones, together with degree of epiphyseal ossification and fusion, are identified and mapped into standardized reference atlases to estimate bone age. Reference atlases represent appearances of the average skeleton for a specific age and gender as acquired from healthy children and adolescents, such as the Tanner and Whitehouse (TW3) method [16,17] and the Greulich and Pyle (G&P) atlas [18], both using hand-wrist radiographs. The TW3 system involves examining the maturation level of each bone separately and combining the grades to produce a total score, which is then matched into a reference table. The G&P atlas presents a single standardized image for a range of ages of each gender. However, racial disparity was not properly considered, and acceleration–deceleration growth patterns failed to be presented in these two bone age atlas methods [19]. Therefore, several simplified skeletal maturity grading systems have been proposed specifically for adolescent idiopathic scoliosis management, such as the olecranon method [20], the Risser sign, Sander’s stage, and distal radius and ulna (DRU) grading [21]. Among these, the DRU grading is a straightforward and reliable method for assessing skeletal maturation, particularly in scoliosis patients [22]. There are 11 ordinal grades for the distal radial physis and 9 ordinal grades for the distal ulnar physis, which are tackled as an image classification problem. The stages in the DRU classification are evenly distributed throughout the pubertal age. Medial capping of the distal radius (R7) and the early appearance of the ulna styloid, with the head of the ulna distinctly defined and denser than the styloid (U5), signify the peak growth spurt. In contrast, blurring of the distal radial growth plate (R11) and fusion of the ulna epiphysis (U9) indicate the cessation of longitudinal growth (Supplementary Figure S1). However, barriers to the outpatient clinical setting remain due to time-consuming and subjective manual assessment. In addition, several scoliosis idiopathic scoliosis prognostic tools have been developed for curve progression risk evaluation to assist treatment, taking manually assessed DRU maturity as risk factors [23,24,25]. The lack of automatic DRU assessment methods limited this prognostic tool’s application in clinical practice.

Computer-aided diagnosis methods have been reported for automatic skeletal maturity assessment, which can be divided into conventional machine learning approaches and deep-learning approaches, according to modeling methodology. The former is generally performed by relying on manually designed visual features from entire images or local informative regions, such as shape, intensities, and texture information of epiphyses regions [26]. BoneXpert is a commercial automated product for bone age assessment based on feature engineering and machine learning methods. It was developed to automatically reconstruct borders of 15 bones from hand X-rays, which were subsequently utilized for feature quantification with principal component analysis (PCA) [27]. Extracted image features, such as bone morphology, intensity, and texture scores, were utilized to implement a unified bone age assessment of TW3 and G&P by mapping functions to give a relative score. Similarly, a content-based image retrieval (CBIR) method was proposed to extract region of interest (ROI) patches from hand X-rays, utilized for bone age assessment, with a combination of cross-correlation, image distortion models, and Tamura texture features [28]. Subsequently, a combination of support vector machine (SVM) and CBIR was proposed for semi-automatic bone age assessment of 14 epiphyseal regions from hand radiographs [29]. The outcomes of these models showed mean absolute errors (MAEs) varying from 10 to 28 months, and these were highly susceptible to the quality of hand-wrist X-ray images. Feature engineering-based methods may not exploit sufficient discriminative information for estimation. Additional manual annotations also limited automatic application in clinic.

Deep learning approaches have the advantage of automating the extraction of imaging features from ROI, which makes it possible to evaluate skeletal maturity using radiographs automatically via data-driven approaches. The majority of automated bone maturity assessment methods use left-hand X-ray scans built upon the TW3 or G&P methods, with big public datasets such as the digital hand atlas database [10] and RSNA bone age dataset [30]. These automatic methods formulated bone age estimation as a regression problem with continued output to minimize error between prediction and ground truth (average of a panel of pediatric radiologists). Bonet was the first study to utilize the convolutional neural network (CNN) for automated bone age estimation based on hand X-rays, achieving an average discrepancy between manual and automatic evaluation of about 0.8 years [31]. An informative region localization method based on unsupervised learning was proposed to estimate bone age without manual annotations [32]. It was implemented on the MobileNet deep structure and achieved an MAE of 6.2 months on the RSNA dataset. A combination of the cascaded critical bone region extraction network and gender-assisted bone age estimation network also was reported to achieve bone age prediction with unsupervised learning [33]. An ensemble learning approach was developed to integrate multiple VGG encoding networks for 13 predefined ROIs of hand radiographs, achieving an MAE of 0.46 years on the TW3 atlas [34]. A vision transformer deep structure was implemented to incorporate whole hand X-rays and extracted ROIs for Fishman’s skeletal maturity grading, achieving a mean absolute error and a root mean square error of 0.27 and 0.604, respectively [35]. CNN variants such as ResNet, VGG-Net, AlexNet, and DenseNet have been reported to be applied in cervical vertebra maturation grading, which contains six maturity stages [36].

By contrast, automatic grading of simplified skeletal maturity has often been defined as a classification task from medical images, generally aiming to facilitate application in a scoliosis management panel. CNN has been reported to be applied for automatic Risser sign classification with pelvic radiographs, achieving 78% overall accuracy [37]. Efficient-Net was implemented to assess Sauvegrain maturity stages [38] automatically on elbow X-rays, with a reported accuracy of 74.5% [39]. Two studies reported application of deep learning for automatic DRU stage grading, using a ResNet model and an ensemble of DenseNet [24,40]. However, both studies were limited to a partial range of bone maturity (R7U6 to R11U9), failing to be applied in accurate estimation of growth acceleration stages in early adolescence. Because their datasets were both collected from scoliosis patient cohorts that were generally accessed after scoliosis screening procedures, patients referred to clinics were approaching maturation. Research gaps also lie in the application of efficient feature encoding methods for accurate grading, especially in distinguishing between adjacent stages, to satisfy clinical application.

The multi-task learning method allows multiple branches of the network to share the detected features among data points of different categories. These task branches represent specific feature maps for attribute categories, and multi-task training on extracted feature maps resulted in attribute inference. Some studies have utilized multi-task learning methods for image classification and segmentation. By introducing a shared encoder between two task branches, joint features of tasks were extracted, which in turn improved prediction performance. For example, a multi-task model was developed for segmentation and classification of breast tumors in 3D ultrasound images; it demonstrated that the multi-task method improves both segmentation and classification over the single-task learning counterparts [41]. The multi-task method was also applied to improve the triple task for chest CT images for segmentation, reconstruction, and pneumonia diagnosis [42].

We hypothesized that improved performance of DRU stage classification resulted from introducing a ROI segmentation branch via a multi-task framework. This is because boundary identification of the distal radius and ulna from hand-wrist radiographs are an important aspect of DRU maturity grading, as assessments of radiologists were conducted based on the morphology of the distal ulna and radius. The segmentations of the ROIs are therefore incorporated in the multi-task network model for DRU stage classification. Incorporating attention mechanisms into the U-Net deep structure represents a recent advancement in segmentation models to suppress irrelevant regions in an input image while highlighting salient features. To this end, this study proposes a multi-task learning framework based upon the Attention-U-Net backbone to integrate a segmentation branch and a classification branch for automatic DRU stage grading. A transfer learning approach was employed to pretrain the Attention-U-Net with a DRU segmentation dataset, utilizing the parameter initialization of shared encoders in multi-task frameworks, which were subsequently trained upon a dataset with paired segmentation and classification labels. The proposed approach outperformed other baseline model settings for both distal radius and distal ulna maturity grading on an independent testing set.

## 2. Materials and Methods

### 2.1. Dataset and Pre-Processing

**Figure 1:**
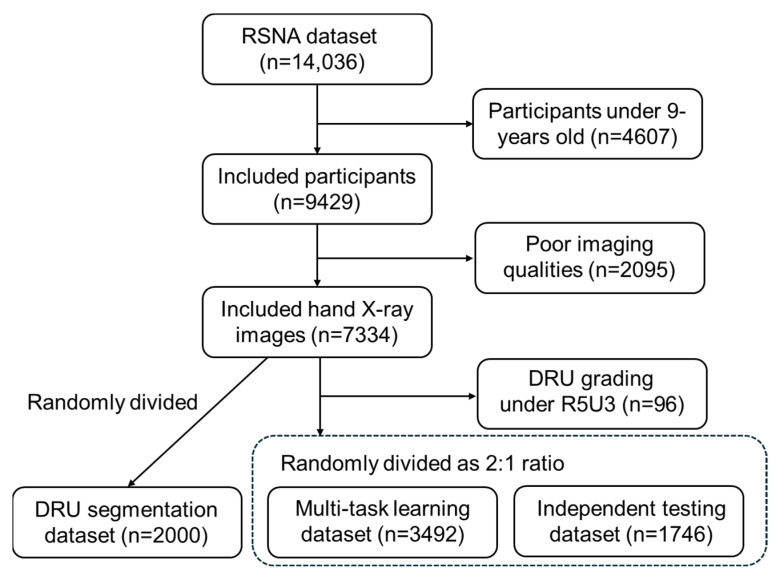
Data inclusion and exclusion flowchart.

The public RSNA pediatric bone age dataset contains 14,036 hand X-ray images of subjects aged between 0 and 19 years old. As our study was aimed at an adolescent cohort, 9429 radiographs of participants between 9 and 19 years old were identified, covering the whole stage from growth spurt to growth cessation for scoliosis progression. Amongst these, 2059 images were excluded due to criteria such as (1) poor imaging quality (over/underexposure) or low image resolution, (2) shrouded wrist regions, and (3) exhibition of bony deformities or non-standard imaging posture; examples of excluded images are shown in Supplementary Figure S2. Images with rotated or contracted hand objects were corrected into the standard front view manually. The remaining 7334 images were randomly separated as a distal radius and ulna segmentation dataset (*n* = 2000), a multi-task dataset (*n* = 3492), and an independent testing set (*n* = 1746). Then, 96 images with a DRU grading below R5U3 were excluded. The data inclusion and exclusion processes are shown in Figure 1.

**Figure 2:**
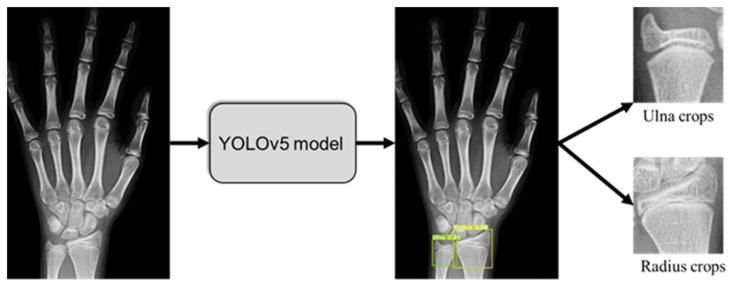
DRU object detection from hand X-rays and crops extraction.

**Figure 3:**
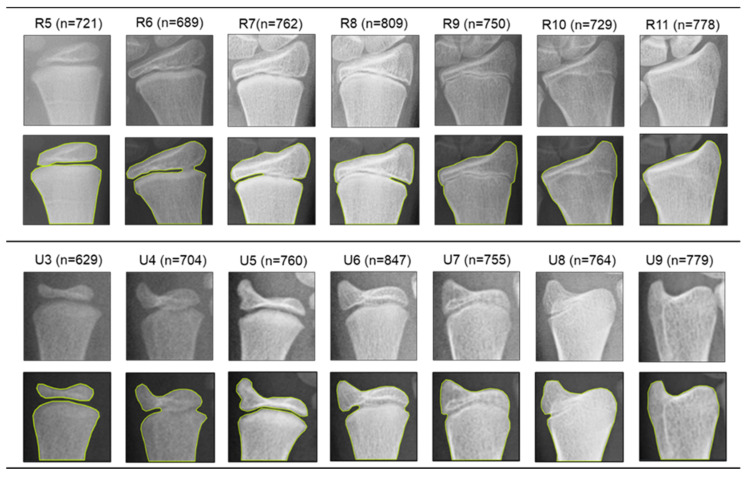
Represented radiographs, segmentation labels, and data size of different ulna and radius maturity stages.

An open-source hand X-ray processing framework based on Yolov5 was employed to detect distal radius and ulna ROIs automatically for all images (Figure 2). As described in our previous study [24], this ROI detection framework implemented a Yolov5m structure that was subsequently trained and validated on 710 hand radiographs labeled by orthopedic surgeons; it can be accessed through github.com/whongfeiHK/AIS-composite-model for public use. Extracted ROIs were resized into 256 × 256 crops via zero-padding operations and saved as single-channel grayscale images in JPG formatting. DRU grades of the multi-task learning dataset and the independent testing set were labeled by two orthopedic surgeons and two radiologists according to assessment protocols [21]. Segmentation labels of the segmentation dataset, multi-task learning dataset, and independent testing dataset were generated by an experienced orthopedic researcher with Roboflow. Examples of labeled images for each maturity stage are shown in Figure 3; DRU radiologic morphology is illustrated in Supplementary Figure S1.

### 2.2. Attention-U-Net as Backbone of Muti-Task Framework

The multi-task learning framework was conducted using Attention-U-Net models that were implemented by introducing attention mechanisms into encoder–decoder deep structures [43]. Fully convolutional image segmentation models represented by U-Net outperformed traditional approaches by combining the benefits of speed and accuracy. This is mainly attributed to the (1) precise localization of object boundaries by integrating low-level and high-level sematic information via skip connections and (2) fully convolutional operations, which allow for effective processing of sizable images and rapid segmentation mask generation. Based on this, an attention mechanism was introduced to suppress the irrelevant information of input images while highlighting the salient features that are passed through the skip connections.

**Figure 4:**
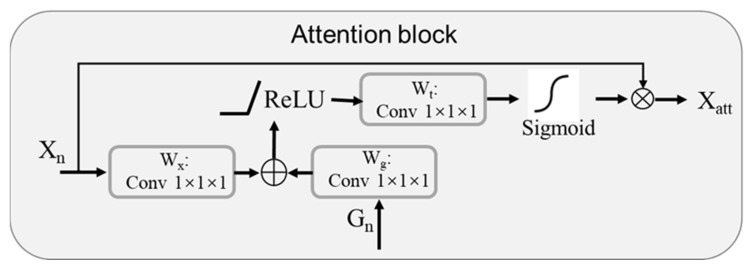
Schematic of the implemented additive attention gate.

The encoder of the implemented model contained five sequentially connected convolution layers followed by max-pooling layers that capture the context and reduce spatial dimensions while increasing the feature channels. This process extracted essential feature representations from input ROI crops, such as edges, textures, and patterns. Symmetrically, a five-layer decoder incrementally increased feature map dimensions through up-sampling operations to combine the high-resolution features from the contracting path, enabling precise localization and restoring the spatial information lost during the encoding process. Additionally, the decoder employed convolutional layers to refine the feature maps and generate a segmented mask. As shown in Figure 4, an attention gate is proposed to focus on targeted regions of feature maps while suppressing feature activations in irrelevant regions. Input features $X^{l}$ are adjusted using attention coefficients $\alpha_{i}^{l}$ with element-wise multiplication, which can be formulated as

(1) $$x_{att}^{l}=\alpha_{i}^{l}\cdot x_{i}^{l}$$

The selection of spatial regions involves analyzing both the activations and the contextual information provided by the gating signal $g^{l}$, obtained from a coarser scale. The overall process of attention coefficient $\alpha_{i}^{l}$ calculation can be formulated as

(2) $$q_{att}^{l}=\psi^{T}\left(\sigma_{1}\left(W_{x}^{T}x_{i}^{l}+W_{g}^{T}g_{i}+b\right)\right)+b_{\psi }\;$$

(3) $$\alpha_{i}^{l}=\sigma_{2}\left(q_{att}^{l}\left(x_{i}^{l};g_{i};\theta_{att}\right)\right)$$

where $\sigma_{2}$ is the sigmoid activation function, and attention gating is described as a parameter set $\theta_{att}$ that contains linear transformations with $W_{X}$ and $W_{g}$, computed with channel-wise 1×1×1 convolutions for the input tensor.

### 2.3. Multi-Task Learning Framework for DRU Grading

**Figure 5:**
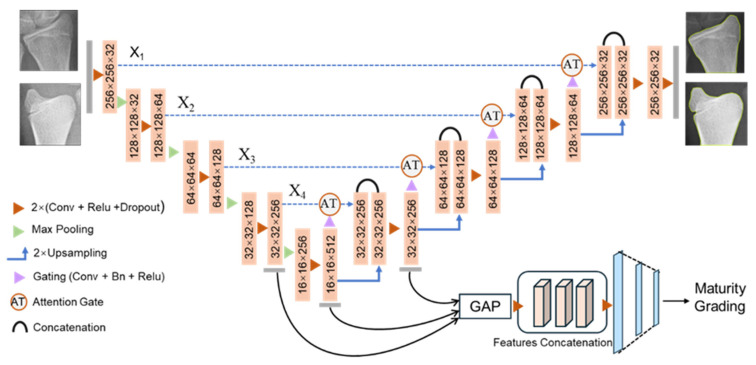
Proposed multi-task learning framework with attention mechanism for DRU maturity classification and segmentation.

The proposed multi-task framework contains an object segmentation module and a classification module, taking Attention-U-Net as backbone network (Figure 5). Two modules shared the same encoder network, with initial parameters transferred from the pretrained Attention-U-Net. Thus, common features for both classification and segmentation were extracted. The segmentation branch was implemented as the decoder section of Attention-U-Net described above. For the maturity stage grading branch, feature maps from the last block of encoder, the bridge, and the first block of decoder were extracted and concatenated for classification. To solve the problem of concatenation between multi-scale feature maps, a global average pooling (GAP) layer was added to the end of each block to resize the feature maps. Subsequently, a recombinant feature map block was connected, with three fully connected layers with dropouts for classification. The first two fully connect layers contained 256 and 128 units and were activated with ReLU function. A final dense layer contained 7 units and was activated with the softmax function to predict 7 stages of the radius (R5–R11) and 7 stages of the ulna (U3–U9) in two separate models.

The dice coefficient loss was utilized for the loss function of the segmentation branch, which was formulated as

(4) $$L_{seg}=D_{loss}=1-\frac{2\times \left|X\cap Y\right|+\theta }{\left|X\right|+\left|Y\right|+\theta }$$

where *X* and *Y* denote mask matrixes of prediction and ground truth, and *θ* is a smoothing factor to avoid division by zero.

We employed cross-entropy loss to optimize the classification branch, which is described as

(5) $$L_{cla}=-\frac{1}{n}\overset{n}{\underset{i=1}{\sum }}\overset{7}{\underset{j=1}{\sum }}\left(y_{i,j}\cdot \log\left(p_{i,j}\right)\right)$$

where *n* is the number of cases, $y_{i,j}$ are the ground truth for class j of an instance i, and $p_{i,j}$ is the predicted probability for class j for instance i. Thus, for the proposed multi-task framework, the combined loss function for union training is

(6) $$L_{m}=\lambda L_{seg}+\left(1-\lambda \right)L_{cla}$$

where $L_{m}$ is the multi-task loss, and λ∈ [0, 1] is a hyperparameter to determine the weight of tasks. Experiments indicated that the model with λ=0.4 achieved the best classification performance.

### 2.4. Model Training and Evaluation

A transfer learning strategy was proposed for multi-task model training. Firstly, the Attention-U-Net backbone was pretrained, tuned and tested on the DRU segmentation dataset with a 7:1:2 ratio division. The pretrained parameter set of the Attention-U-Net encoder was transferred into the shared encoder of the multi-task model as parameter initialization. Subsequently, the multi-task learning model was trained on the multi-task dataset containing both classification and segmentation labels as described above. A five-fold cross-validation method was utilized for model training and optimal hyperparameter set searching. We conducted data augmentation for each training fold of cross-validation with the flip horizontal method. All models were implemented using Pytorch frameworks based on Python 3.8. The Adam optimizer algorithm was employed with a batch size of 16 and a decaying learning rate initialized at 0.005 for gradient updates. The training process was conducted on a computer server equipped with two NVIDIA Tesla T4 GPUs and 128 GB RAM.

Performance evaluation was conducted on the independent testing set. Quantitative evaluation of the distal radius and ulna segmentation was evaluated by IoU (Intersection Over Union) and the Dice similarity coefficient (*DSC*) in comparison to manually generated ground truths. The matrixes were formulated as follows:

(7) $$IoU=\frac{\left|X\cap Y\right|}{\left|X\cup Y\right|}$$

(8) $$DSC=\frac{2\times \left|X\cap Y\right|}{\left|X\right|+\left|Y\right|}$$

where *X* and *Y* denote mask matrixes of prediction and ground truth. The classification branch of multi-task learning for skeletal maturity grading was evaluated via measures of average accuracy, precision, recall, and F1-score for each DRU stage. Five-times repeated model training processes with an optimal hyper-parameter set followed by bootstrap sampling (*n* = 5000) on an independent testing dataset were employed to generate 95% confidence intervals.

### 2.5. Baseline Model Setting for Performance Comparison

Several baseline deep learning models and multi-task framework settings were implemented, trained, and tested on our dataset for comparison with our proposed approach.

(1) An ensemble-based DenseNet framework that integrated five independent DenseNet models with different model configure settings [40]. This was the first report utilizing a deep learning method for automatic DRU maturity grading but was only limited to four stages of the distal radius and three stages of the distal ulna. We implemented their framework and fine-tuned it on our dataset for performance evaluation.

(2) ResNet models based on regression problem definition with continuous output. The DRU grading estimates were attained as numerical outputs rounded to the nearest integer [24]. This model took radius and ulna ROI crops as input without segmentation. We conducted experiments on both ROI crops and segmented images.

(3) We also implemented a two-stage assessment framework consisting of segmentation and subsequent classification. The distal radius and ulna segmentations were performed with Attention-U-Net as described above. The Attention-U-Nets were trained upon the segmentation dataset and then applied to generate segmented images of the classification dataset and independent testing set. Efficient-Net B0 to B7 structures [44] were implemented as classifiers for performance comparison.

(4) To investigate the advantages of the attention mechanism in the proposed multi-task framework, we replaced the Attention-U-Net backbone as conventional U-Net configured with the same layers and hyperparameters.

(5) To investigate the effectiveness of the proposed encoder pretraining and parameter transferring method, we neglected the process of Attention-U-Net pretraining upon the separate segmentation dataset. The multi-task framework was directly trained based on paired segmentation and grading labels with random parameter initialization.

(6) As suggested in [24], we also formulated the proposed framework as a regression problem with continuous output. The last layer of the classification branch was replaced with a single unit with a ReLU activation function. The prediction outputs were then attained as a numerical value rounded to the nearest integer.

## 3. Results

The independent testing set containing 1746 hand X-rays was utilized for the proposed method and baseline model evaluation. ROI crops of the ulna and radius were generated with the pretrained Yolov5 tool. The radius set for testing contained 245 images as grade R5, 251 images as grade R6, 237 images as grade R7, 219 images as grade R8, 272 images as grade R9, 246 images as grade R10, and 276 images as graded R11. The ulna set consisted of 237 images as grade U3, 222 images as grade U4, 261 images as grade U5, 255 images as grade U6, 273 images as grade U7, 246 images as grade U8, and 252 images as grade U9. DRU maturity grading performance was evaluated on the proposed multi-task framework in comparison to the other six baseline models, as described above. The mean values for model performance following five repeated experiments and bootstrap sampling are summarized in Table 1 and Table 2 for distal radius and ulna grading, respectively.

Regarding the two previously published models, the ensemble of DenseNet [14] demonstrated an accuracy of 86.2% (85.4–88.7%), precision of 87.2% (85.9–87.7%), recall of 85.3% (84.4–86.2%), and F1-score of 86.2% (85.1–86.9%) for radius maturity stage classification, as well as an accuracy of 83.4% (80.9–84.1%), precision of 81.3% (79.6–83.0%), recall of 83.9% (82.1–84.4%), and F1-score of 83.2% (81.5–84.0%) for ulna classification. ResNet applied on the regression formulation [15] achieved an accuracy of 83.3% (81.8–84.6%), precision of 84.2% (83.0–85.4%), recall of 82.6% (81.1–83.0%), and F1-score of 83.4% (82.0–84.2%) for radius assessment, while it reported an accuracy of 81.0% (79.5–83.0%), precision of 78.6% (77.9–80.4%), recall of 81.5% (80.1–82.4%), and F1-score of 80.8% (79.5–81.9%) for ulna assessment.

The Efficient-Net B4 model outperformed other Efficient structures of B0 to B7, achieving an accuracy of 84.5% (82.2–85.6%), precision of 83.9% (82.8–84.5%), recall of 85.2% (84.1–86.3%), and F1-score of 84.5%(83.4–85.4%) for the radius stage, as well as an accuracy of 82.8% (81.7–83.6%), precision of 83.9% (82.0–84.7%), recall of 82.1% (81.5–83.9%), and F1-score of 82.5% (81.6–84.1%) for ulna classification. Incorporating an additional segmentation module with a deep classifier improved prediction performance. Efficient-Net B4 for segmented ROI objects achieved an accuracy of 87.3% (86.0–88.4%), precision of 86.8% (86.3–88.2%), recall of 88.5% (83.3–88.9%), and F1-score of 87.6% (84.3–88.5%) for radius classification, as well as an accuracy of 85.6% (84.1–85.9%), precision of 86.0% (84.4–86.7%), recall of 83.2% (83.0–84.5%) and F1-score of 83.9% (83.3–85.0%) for ulna classification. This indicated that boundary information extraction was efficient for DRU maturity assessments.

The multi-task learning with U-Net as the backbone presented more limited performance than the proposed method, achieving an accuracy of 89.4% (88.2–91.2%), precision of 90.3% (88.1–92.0%), recall of 88.0% (87.4–90.8%), and F1-score of 89.1% (87.7–91.4%) for radius classification, as well as an accuracy of 85.9% (84.3–86.7%), precision of 85.0% (83.9–86.2%), recall of 86.7% (84.9–87.0%), and F1-score of 86.3% (84.6–86.8%) for ulna classification. The findings demonstrated that the attention mechanism improved feature encoding efficiency by focusing on targeted regions of feature maps while suppressing feature activations in irrelevant regions. The multi-task framework without a pretraining process also showed relatively low performance, with an accuracy of 92.5% (90.3–93.1%), precision of 91.4% (89.9–93.0%), recall of 93.3% (91.9–94.0%), and F1-score of 92.3% (90.9–93.5%) for radius classification, as well as an accuracy of 87.2% (86.4–88.6%), precision of 85.0% (83.8–86.2%), recall of 87.9% (86.1–88.5%), and F1-score of 87.2% (85.5–87.9%) for ulna classification. The proposed transfer learning strategy for hard shared encoder parameters improved recognition performance. The setting of regression outputs did not result in much improvement compared to the proposed classification scheme, and only achieved an accuracy of 92.2% (90.7–93.6%), precision of 91.8% (89.3–92.8%), recall of 92.9% (90.0–93.5%), and F1-score of 92.3% (89.6–93.1%) for radius classification, as well as an accuracy of 89.1% (87.0–91.1%), precision of 90.3% (88.7–90.9%), recall of 88.0% (87.6–89.8%), and F1-score of 88.6% (87.9–90.1%) for ulna classification.

**Figure 6:**
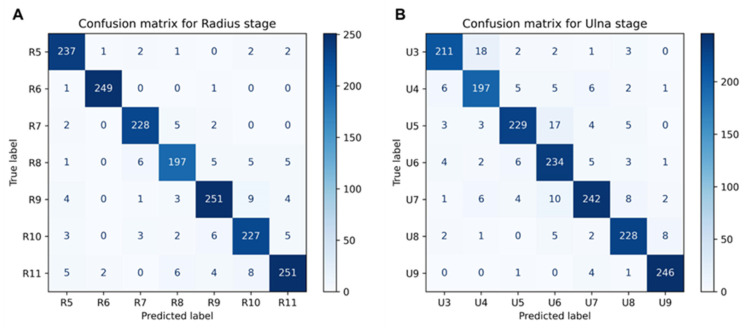
(**A**) Confusion matrix for radius stage grading for proposed method. (**B**) Confusion matrix for ulna stage grading for proposed method.

The proposed multi-task learning framework combining an attention mechanism and transfer learning-based parameter initialization achieved the best performance figures for both distal radius and ulna maturity grading. Model accuracy was 94.3% (91.4–95.0%), precision was 93.8% (90.7–94.3%), recall was 94.6% (92.1–95.2%), and F1-score was 94.2% (91.4–94.7%) for radius maturity classification. The framework achieved an average accuracy of 90.8% (88.6–93.3%), an average precision of 90.3% (89.0–92.6%), an average recall of 92.4% (90.1–94.2%), and an average F1-score of 91.9% (89.8–93.8%) for ulna maturity classification. The corresponding confusion matrixes for the proposed method are shown in Figure 6.

Although DRU object segmentation was a secondary outcome of this study, performance evaluation was also conducted amongst the proposed methods and three baseline methods for the independent testing set (Table 3). The conventional U-Net achieved an IoU of 0.912 (0.906–0.926, 95% CI) and a DSC of 0.930 (0.915–0.939) for distal radius segmentation and an IoU of 0.918 (0.897–0.922) and a DSC of 0.920 (0.908–0.934) for distal ulna segmentation. By comparison, the multi-task learning framework improved segmentation performance, with 0.937 (0.912–0.944) IoU and 0.943 (0.921–0.949) DSC for the radius, as well as 0.918 (0.897–0.922) IoU and 0.937 (0.919–0.945) DSC for ulna segmentation. Similarly, introducing attention gating into the U-Net structure achieved an improved segmentation and demonstrated an IoU of 0.945 (0.932–0.953) and a DSC of 0.950 (0.939–0.958) for radius segmentation, as well as an IoU of 0.948 (0.933–0.961) and a DSC of 0.950 (0.937–0.962) for ulna segmentation. The proposed framework incorporating both the attention mechanism and multi-task learning achieved the best performance and reported an IoU of 0.960 (0.951–0.973) and a DSC of 0.973 (0.962–0.979) for radius segmentation, as well as an IoU of 0.966 (0.948–0.977) and a DSC of 0.969 (0.962–0.978) for ulna segmentation. The results demonstrated that introducing classification information improved segmentation performance, although segmentation is not a primary objective in adolescent skeletal maturity assessments.

## 4. Discussion

Skeletal maturity assessment in children and adolescents plays an important role in the management of growth-related diseases such as scoliosis and hormonal disorders [9]. In particular, numerous investigations on adolescent idiopathic scoliosis emphasize the significance of growth and the swift advancement of the spinal curve during the peak of the growth spurt [45,46]. Understanding the patient’s growth potential and reaching the near end-stage of growth is crucial for prognosis. It guides the treating physician in determining the appropriate treatment approach, including the observation interval, timing for starting bracing therapy, when to stop bracing, and the timing of instrumentation and surgery fusion.

The Risser sign and menarche age had been reported to show weak correlation with peak height velocity and no ability to predict growth cessation, as these appear after the peak of the adolescent growth spurt [4,47]. The TW3 and G&P provide comprehensive methods of quantifying bone age via observing the degree of epiphyseal ossification and fusion in hand radiographs, which allows more accurate prediction of bone maturation. However, assessments of all finger digits and DRU epiphysis features make these schemes time-consuming and challenging to implement in an outpatient clinical setting. Progression stages of the DRU physes can be a simplified index, as it encompasses the entire period of skeletal growth and is the last to close [21]. Nonetheless, Sanders et al. [47] reported DRU epiphysis in the TW3 method shows very limited correlations with growing peaks. Because the DRU in the TW3 methods were initially designed to be used alongside the epiphysis of the finger phalanges and possess a wide interval between each stage, which are not accurate for predicting growth spurts. By contrast, the DRU grading scheme identified additional stages for the DRU that are more evenly distributed throughout the pubertal phase, with each stage having a bone age gap interval of one year. Growth spurt peak was observed at radiologic stages R7 and U5, characterized by the medial capping of the distal radius epiphysis and the appearance of the ulna styloid in the ulna epiphysis, whereas blurring of the distal radial growth plate (R11) and fusion of the ulna epiphysis (U9) indicate the cessation of longitudinal growth [48,49]. This grading scheme has been validated to closely correlate with the adolescent growth spurt and the cessation of growth in scoliosis patients [50]; thus, it may provide valuable insights in clinical management options.

Besides the DRU grading scheme analyzed in this paper, some other studies have also investigated correlations between DRU maturity and hand-wrist radiographs. Sallam et al. [51] analyzed the geometric development of the wrist in relation to the changes in its ossification pattern via a retrospective multicenter study of 896 children. This study determined that radiologic parameters of the ulna and radius exhibited consistent anatomic changes before the 12-year-old time-point. Huang et al. [52] proposed a modified wrist skeletal maturity system incorporating an epiphyseal and metaphyseal ratio of five physes: the distal radius, distal ulna, and 1, 3, and 5 metacarpals, together with ulnar styloid height and radial styloid height. When combined with chronological age and sex parameters, the system achieved an improved skeletal maturity estimation compared with the G&P method. Recently, a modified Fels wrist skeletal maturity system containing eight anteroposterior wrist radiographic parameters was developed to quantify adolescent skeletal maturity [53]. The modified Fels was reported to provide more accurate, reliable, and rapid skeletal maturity estimation than G&P and Sander’s stage systems. These three simplified wrist maturity systems were implemented by quantifying radiographic parameters, whereas the DRU grading scheme analyzed in this paper used maturity stage classification via observation of the radius and ulna morphology. The prognostic value of these three latest wrist maturity systems in scoliosis clinical management have not been investigated, and computer-aided assessment methods have not been developed.

Computer-aided skeletal maturity prediction tools aim at fast and accurate assessments via machine learning methods to promote clinical practice. The majority of studies focus on automated TW3 or G&P bone age predictions for hand X-rays. An essential advance of bone age assessment study is the application of a CNN-based computer vision model to learn key features from radiographs automatically, without the need for complex feature engineering and ROI extraction, such as Bonet for a TW3 bone age prediction [31] and a CNN model for G&P bone age prediction [54]. Concerning distal radius and ulna maturity assessment, both were previously reported using ensemble densely connected CNN [40] and residual regression network [24] convolutional operations for DRU ROI crops for maturity feature identification and expression. Specifically, the ensemble dense CNN integrated multiple classifiers with different hyperparameter settings via a voting mechanism to improve the prediction performance of single classifiers [40]. The residual neural network with continued DRU grading output reported higher accuracy than classification problem definition [24].

Skeletal maturities were discriminated by morphology discrepancy at individual growing stages of characteristic bones, such as the distal radius and ulna [55]. Identification of the maturity stages improved accurate boundary recognition of characteristic bones, which highlighted morphological differences and in turn facilitated maturity classification. This formed the basis for multi-task learning approaches combining segmentation and classification modules with a hard shared encoder. An attention mechanism was introduced to suppress irrelevant regions of inputs while highlighting salient features [56,57]. Pretraining and parameter transferring reduced the requisite computational costs for multi-task framework initialization. A limitation to this study is that prediction error may occur when morphological features were between two adjacent stages, since X-ray changes were graduated. Thus, a previous study [24] suggested a regression method to generate continued output, but this approach did not perform well on our dataset. Establishing a sequential dataset of X-rays at different growing stages may improve prediction performance, which can integrate dynamic morphology changes into a spatial-temporal prediction model. Our platform promises accurate DRU stage grading automatically, with an average accuracy over 90%, outperforming previously reported models [24,40]. Additionally, our model can assess seven stages of both radius and ulna maturity, covering the integral puberty skeletal growing period. The proposed framework promises to be integrated with advanced scoliosis prognostic tools [23,24,48] to facilitate progression risk evaluation and personalized management.

## 5. Conclusions

We demonstrated that a multi-task learning framework could achieve accurate assessment of DRU skeletal maturity. This framework utilized an Attention-U-Net as the backbone, connecting a dense network as a classifier via a shared encoder. The proposed model was trained and tested on an RSNA dataset. Our model has the potential to be integrated into scoliosis prognostics tools directly to facilitate personalized diagnostics and management, representing a substantial advancement in child and adolescent healthcare.

## Figures and Tables

**Table 1:** Performance comparison of proposed method and baseline models for distal radius maturity grading.

Models	Accuracy (95%CI)	Precision (95%CI)	Recall (95%CI)	F1 score (95%CI)
Ensemble DenseNet [40]	86.2% (85.4–88.7%)	87.2% (85.9–87.7%)	85.3% (84.4–86.2%)	86.2% (85.1–86.9%)
ResNet [24]	83.3% (81.8–84.6%)	84.2% (83.0–85.4%)	82.6% (81.1–83.0%)	83.4% (82.0–84.2%)
Efficient-Net B4	84.5% (82.2–85.6%)	83.9% (82.8–84.5%)	85.2% (84.1–86.3%)	84.5% (83.4–85.4%)
Two-stage framework	87.3% (86.0–88.4%)	86.8% (86.3–88.2%)	88.5% (83.3–88.9%)	87.6% (84.3–88.5%)
U-Net with multitask model	89.4% (88.2–91.2%)	90.3% (88.1–92.0%)	88.0% (87.4–90.8%)	89.1% (87.7–91.4%)
Multi-task without pretrain	92.5% (90.3–93.1%)	91.4% (89.9–93.0%)	93.3% (91.9–94.0%)	92.3% (90.9–93.5%)
Multi-task with regression	92.2% (90.7–93.6%)	91.8% (89.3–92.8%)	92.9% (90.0–93.5%)	92.3% (89.6–93.1%)
Proposed method	94.3% (91.4–95.0%)	93.8% (90.7–94.3%)	94.6% (92.1–95.2%)	94.2% (91.4–94.7%)

**Table 2:** Performance comparison of proposed method and baseline models for distal ulna maturity grading.

Models	Accuracy (95%CI)	Precision (95%CI)	Recall (95%CI)	F1 score (95%CI)
Ensemble DenseNet [40]	83.4% (80.9–84.1%)	81.3% (79.6–83.0%)	83.9% (82.1–84.4%)	83.2% (81.5–84.0%)
ResNet [24]	81.0% (79.5–83.0%)	78.6% (77.9–80.4%)	81.5% (80.1–82.4%)	80.8% (79.5–81.9%)
Efficient-Net B4	82.8% (81.7–83.6%)	83.9% (82.0–84.7%)	82.1% (81.5–83.9%)	82.5% (81.6–84.1%)
Two-stage framework	85.6% (84.1–85.9%)	86.0% (84.4–86.7%)	83.2% (83.0–84.5%)	83.9% (83.3–85.0%)
U-Net with multitask model	85.9% (84.3–86.7%)	85.0% (83.9–86.2%)	86.7% (84.9–87.0%)	86.3% (84.6–86.8%)
Multi-task without pretrain	87.2% (86.4–88.6%)	85.0% (83.8–86.2%)	87.9% (86.1–88.5%)	87.2% (85.5–87.9%)
Multi-task with regression	89.1% (87.0–91.1%)	90.3% (88.7–90.9%)	88.0% (87.6–89.8%)	88.6% (87.9–90.1%)
Proposed method	90.8% (88.6–93.3%)	90.3% (89.0–92.6%)	92.4% (90.1–94.2%)	91.9% (89.8–93.8%)

**Table 3:** Segmentation performance comparison of proposed methods and baseline settings.

Models	Distal Radius		Distal Ulna	
	IoU (95% CI)	DSC (95% CI)	IoU (95% CI)	DSC (95% CI)
U-Net	0.912 (0.906–0.926)	0.930 (0.915–0.939)	0.918 (0.897–0.922)	0.920 (0.908–0.934)
Multi-task with U-Net	0.937 (0.912–0.944)	0.943 (0.921–0.949)	0.931 (0.906–0.937)	0.937 (0.919–0.945)
Attention-U-Net	0.945 (0.932–0.953)	0.950 (0.939–0.958)	0.948 (0.933–0.961)	0.950 (0.937–0.962)
Proposed methods	0.960 (0.951–0.973)	0.973 (0.962–0.979)	0.966 (0.948–0.977)	0.969 (0.962–0.978)

## Data Availability

All raw data used in the study are collected from the RSNA public dataset, accessed from https://www.rsna.org/rsnai/ai-image-challenge/rsna-pediatric-bone-age-challenge-2017 (accessed on 21 November 2024). Post labeled dataset and source codes are available from the corresponding author upon reasonable request.

## Supplementary Materials

The following supporting information can be downloaded at: https://www.mdpi.com/article/10.3390/tomography10120139/s1, Figure S1: Distal radius and ulna (DRU) classification of radiologic morphology and its similarity to original TW3 stages; Figure S2: Examples of excluded hand X-ray images of RSNA dataset in this study.
