# Supplementary material for: Automated Distal Radius and Ulna Skeletal Maturity Grading from Hand Radiographs with an Attention Multi-Task Learning Method
Source: Tomography. 2024 Nov 28;10(12):1915–29. doi: 10.3390/tomography10120139 (PMC11679689; doi:10.3390/tomography10120139)

Supplementary Figure S1. Distal radius and ulna (DRU) classification of radiologic morphology and its similarity to original TW3 stages [21].

DRU grading	TW3	Characterization of stages	Radiograph
R5	F	The proximal border of the epiphysis is visible as an irregular, thickened white line, more pronounced on the medial side. The proximal border has a concave shape, and the width of the epiphysis is narrower than that of the metaphysis.	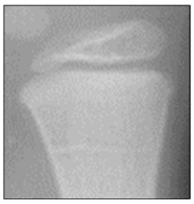
R6	G	Either the medial part of the epiphysis overhangs the metaphysis, the lateral border is wider than the metaphysis, or both. Additionally, the medial border forms an articulation with the ulna	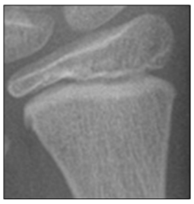
R7	H	The epiphysis is capped on the medial side, while the lateral side is rounded.	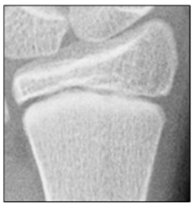
R8	H	Squaring or capping is observed on the lateral proximal corner, and the physeal plate is still clearly visible. The medial and lateral ends of the plate are wider than the center.	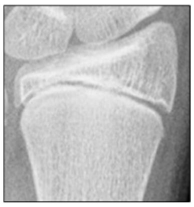
R9	I	The epiphysis is strongly capping the metaphysis, with sclerosis of the physeal space. The growth plate is visible but blurred, and the epiphysis nearly touches the metaphysis at the medial and lateral ends.	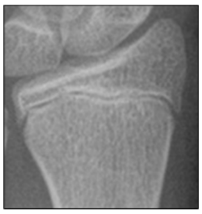
R10	I	The growth plate is completely obliterated, forming a sclerotic line that sometimes appears as a broken line. A notch is visible at either the medial or lateral end of the growth plate.	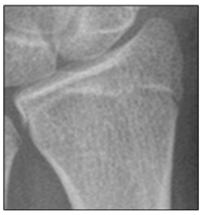

R11	I	Complete fusion is present, with the lateral and medial edges of the physeal line rounded. The growth plate scar may still be visible.	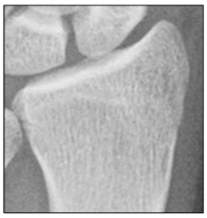
U3	D	The epiphysis is at least half the width of the metaphysis.	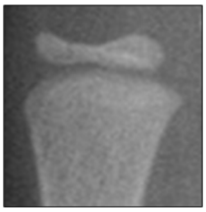
U4	E	The styloid is noticeable on the medial end of the epiphysis.	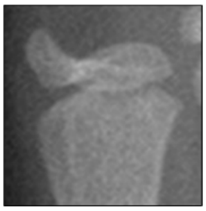
U5	F	The head of the ulna is distinctly defined and denser than the styloid, with the border adjacent to the radial epiphysis being flattened	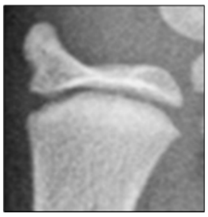
U6	G	The epiphysis is as wide as the metaphysis, and the proximal border of epiphysis overlaps with the metaphysis at the central third.	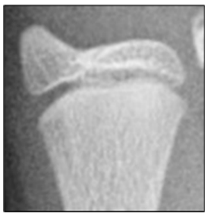
U7	H	Narrowing of the medial physeal plate is observed, with the medial border of the epiphysis and the metaphysis forming a smooth curved line (articulation with the radius). Fusion may be seen on the medial half	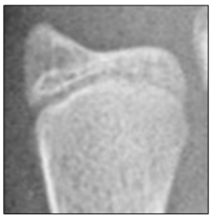
U8	H	An unfused growth plate is visible proximal to the styloid process. In a rotated film, the medial and lateral borders appear fused, but the physeal space can be seen beneath the styloid.	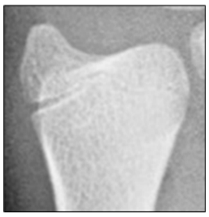

U9	H	Complete fusion	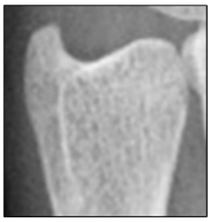

Supplementary Figure S2. Examples of excluded hand X-ray images of RSNA dataset in this study.

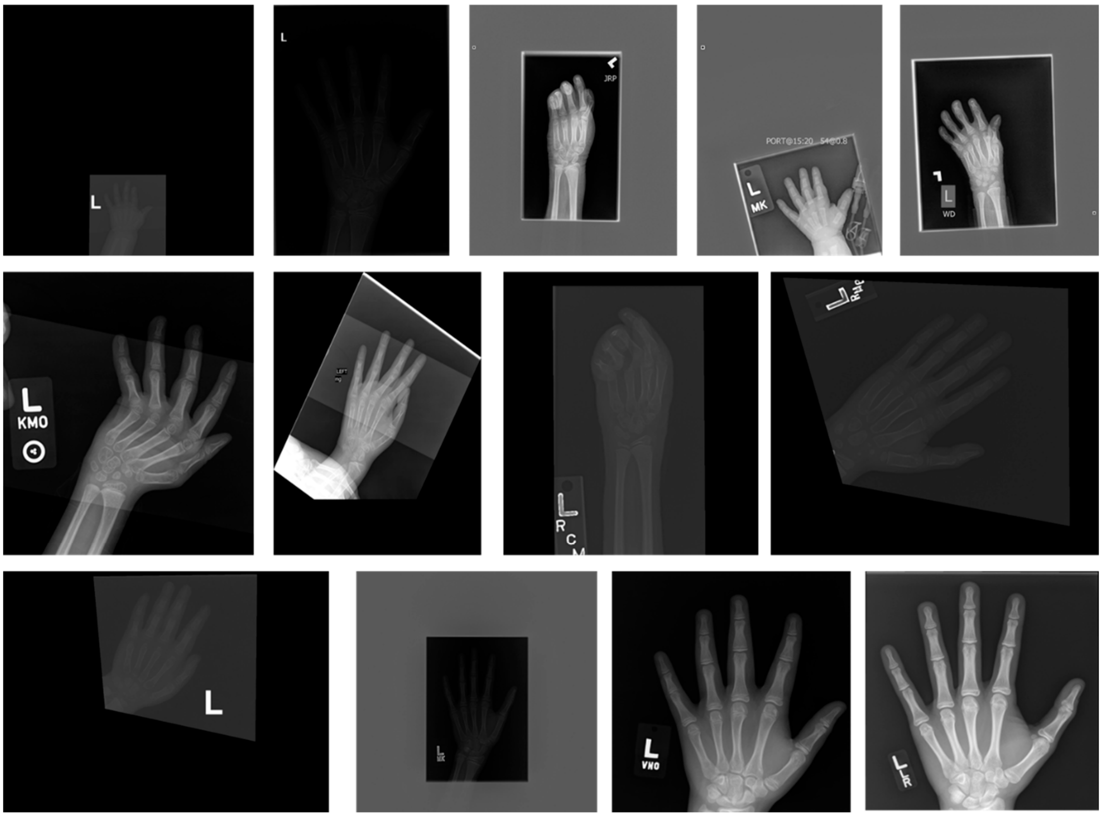

Supplement: Supplementary file 1 [file tomography-10-00139-s001.zip › tomography-3279246-supplementary.pdf]
